# Evaluation of Patient-Reported Outcome Differences by Radiotherapy Techniques for Bone Metastases in A Population-Based Healthcare System

**DOI:** 10.3390/curroncol29030167

**Published:** 2022-03-18

**Authors:** Robert A. Olson, Vincent LaPointe, Alex Benny, Matthew Chan, Shilo Lefresne, Michael McKenzie

**Affiliations:** 1 BC Cancer, Prince George, Prince George, BC V2M 7E9, Canada; 2 BC Cancer, Vancouver, Vancouver, BC V6T 1Z4, Canada; vlapoint@bccancer.bc.ca (V.L.); matthew.chan@bccancer.bc.ca (M.C.); slefresne@bccancer.bc.ca (S.L.); mmckenzi@bccancer.bc.ca (M.M.); 3 Department of Medicine, University of British Columbia, Vancouver, BC V6T 1Z4, Canada; alex.benny@bccancer.bc.ca

**Keywords:** patient-reported outcomes, radiotherapy, bone metastases, stereotactic ablative radiotherapy, intensity-modulated radiotherapy

## Abstract

We assessed whether advanced RT techniques were associated with differences in patient-reported outcomes (PROs). Patients with bone metastases who completed the brief pain inventory (BPI) before and after RT were identified, and RT technique was categorized as simple (e.g., parallel opposed pair) or advanced (e.g., 3D-conformal RT (3DCRT), intensity-modulated RT (IMRT), or stereotactic ablative RT (SABR)). Pain response and patient-reported interference on quality of life secondary to pain was compared. A total of 1712 patients completed the BPI. From 2017–2021, the rate of advanced RT technique increased significantly (*p* < 0.001; 2.4%, 2.4%, 9.7%, 5.5%, 9.3%), with most advanced techniques consisting of IMRT, and only 7% of advanced techniques were SABR. Comparing simple vs. advanced technique, neither the complete pain response (12.3% vs. 11.4%; *p* = 0.99) nor the partial pain response (50.0% vs. 51.8%; *p* = 0.42) was significantly different. There was no significant patient-reported difference in pain interfering with general activity, mood, walking ability, normal work, relationships, sleep, or enjoyment of life. Given that there is increasing utilization of advanced RT techniques, there is further need for randomized trials to assess their benefits given the increased cost and inconvenience to patients.

## 1. Introduction

Bone metastases (BoM) are common, most often resulting from primary prostate, breast, or lung malignancies [1]. Radiotherapy (RT) is an effective technique for palliation, and the topic of multiple randomized controlled trials [1,2,3]. Historically, RT for BoM has been given with simple unplanned radiotherapy (SUPR), such as parallel opposed pairs or single direct fields, though more recently advanced techniques, including 3D conformal radiotherapy (3DCRT), intensity modulated radiotherapy (IMRT), and stereotactic ablative radiotherapy (SABR), are now used more frequently with the topic of randomized trials [4,5,6,7].These advanced techniques are more costly as they require planning time from dosimetrists and medical physicists and are usually associated with a longer time from CT simulation to treatment for patients, potentially leading to a longer time with untreated pain [8,9]. However, advanced techniques have the potential advantage of minimizing toxicity by sparing normal tissue (e.g., with IMRT) or by escalating the dose and offering higher rates of control, or possibly even acting as a cure (e.g., with SABR) [6,10,11,12,13,14]. For example, the phase 2/3 randomized controlled trial SC.24 showed a superior complete pain response with SABR 24 Gy in two fractions over conventional simple external beam radiotherapy at a dose of 20 Gy in five fractions [6], while the landmark SABR-COMET trial has demonstrated a potential overall survival benefit to SABR over the standard of care in patients with oligometastatic disease [10,11,12,13,14]. Both of these trials potentially have limited generalizability, as all clinical trials may, given the selection of fit patients that meet stringent eligibility criteria. Therefore, population-based studies may complement the findings of the randomized trials and provide more generalizable results.

In British Columbia (BC), Canada, all RTs are given at BC Cancer, which is provincially coordinated and population-based [1,15]. In addition, BC’s healthcare is publicly funded, radiation oncologists are on salary, and there is no incentive to prescribe more advanced RT techniques. Since 2013, we have been collecting patient-reported outcomes (PROs) for patients with bone metastases before and after RT at five of six BC Cancer centres, initially with a homegrown questionnaire, and a switch to the Brief Pain Inventory (BPI) in 2017 [16,17]. BC is therefore uniquely situated to perform population-based assessment of PRO changes associated with RT use.

The primary objective of this analysis was to assess whether there is a correlation between simple vs. advanced RT techniques for BoM and PRO pain response and pain interference on quality of life (QoL).

## 2. Materials and Methods

From 2017–2021 inclusive, patients with BoM who completed the BPI PRO questionnaire at one of five BC Cancer centres before (usually at CT simulation) and after RT (vast majority at 6 weeks post last fraction) were identified, and RT technique was categorized as simple (e.g., parallel opposed pair) or advanced (e.g., 3DCRT, IMRT, or SABR). The BPI is a validated tool recommended for use in research assessing pain responses of patients receiving palliative RT [3]. Partial and complete pain response was defined as recommended by international consensus [3]. Pain response was compared using chi-square tests. Patient-reported interference on quality of life (QoL) secondary to pain was compared with t-tests. Multivariable analyses of pain response and pain impact on QoL were compared with logistic and linear regression, respectively.

This study was approved by the joint UBC and BC Cancer Research Ethics Board (protocol code H22-00079 approved 1 January 2022).

## 3. Results

Patient characteristics are presented in Table 1.

**Figure 1:**
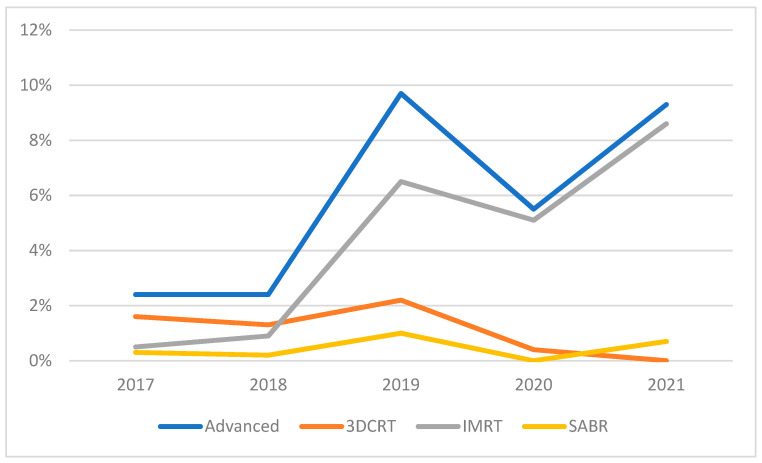
Use of advanced RT techniques over study duration.

The use of advanced RT techniques increased significantly from 2017 to 2021, averaging 5% over the study duration (Figure 1). Most advanced RT techniques were 3DCRT (26%) or IMRT (67%), with only 8% of advanced cases representing SABR. Because of the small SABR and 3DCRT numbers, we performed analyses separately and with all three advanced techniques combined.

Baseline pain by technique did not differ (2.5 vs. 2.6; simple vs. advanced; *p* = 0.58) at baseline. As shown in Table 2, there was no difference in partial or complete pain response by radiotherapy technique on univariable analysis. Age was associated with complete pain response, while primary histology was associated with a partial pain response (Table 2).

After controlling for gender, age, primary histology, and treatment region, there was no significant association of RT technique and either partial (Table 3) or complete (Table 4) pain response. Likewise, there was no significant associations when performing the multivariable analysis looking at partial response with technique divided into simple RT (reference) versus 3DCRT (OR = 1.08; 95% = CI 0.46–2.56; *p* = 0.86), IMRT (OR = 1.02; 0.49–2.09; *p* = 0.97), or SABR (OR = 0.64; 0.10–4.12; *p* = 0.64). Similarly, there was no significant association on multivariable analysis looking at complete response for simple RT (reference) versus 3DCRT (OR = 0.74; 0.21–2.58; *p* = 0.63), IMRT (OR = 1.29; 0.52–3.23; *p* = 0.59), or SABR (OR = 1.16; 0.13–10.36; *p* = 0.89).

RT technique was also not associated with pain’s impact on QoL upon either univariable or multivariable analysis (Table 5; all *p* values > 0.1). Similarly, there were no significant associations on univariable or multivariable analyses when technique was divided into simple RT, 3DCRT, IMRT, and SABR, though numbers were small in multiple categories.

## 4. Discussion

This population-based analysis of a publicly funded RT program, where salaried radiation oncologists have no incentives to prescribe more advanced RT techniques, confirmed a continued increase in the use of advanced RT techniques for BoM from the previously published trend [4]. However, there was no association between RT technique and pain response or pain interference with QoL. Given the vast majority of the advanced RT techniques used in our setting were 3DCRT or IMRT (rather than 7% SABR), the difference compared to simple techniques is predominantly volume (rather than dose), and therefore, we believe interpretation should be focused on patient-reported QoL outcomes (rather than pain response). These findings are important as advanced RT techniques are more costly and adopted more frequently globally without level 1 evidence [9], suggesting more evidence is needed before continued wider adoption of more conformal, advanced RT techniques, especially outside of the oligometastatic setting [10].

Our finding of increased use of advanced RT techniques is consistent with other jurisdictions [5,18]. For example, a recent Australian publication identified a similar increased use of advanced RT techniques, also predominantly IMRT rather than SABR [5]. Our finding of similar pain interference on QoL is also consistent with findings from a randomized phase II from the Netherlands, where they identified that QoL improved similarly in both conventional RT and SABR arms [19].

A recently completed Canadian and Australian SC.24 randomized phase III trial showed a significantly higher complete pain response from SABR compared to conventional RT [6]. However, as mentioned above, our study lacked sufficient SABR numbers to compare pain response from SABR vs. simple conventional RT, and therefore should not be directly compared. Our study was more adequately powered to identify differences in patient-reported QoL, but failed to do so, possibly because the BPI’s measurement of pain’s impact on QoL was too generic. We propose that studies need to focus on QoL specific to the body parts where toxicity is trying to be minimized, such as the SUPR-3D phase III randomized controlled trial, which is focusing on QoL related to nausea and vomiting in patients where IMRT is being assessed in BoM in the lumber spine and pelvis [7]. Our study does add to the literature, as it is a broader, generalizable assessment of pain response and QoL in standard palliative patients receiving RT, rather than the well-selected, fit patients in randomized trials, limited specific subsites (e.g., spinal metastases for SC.24 [6]), and oligometastases (e.g., SABR-COMET [10,11,12,13,14]).

Our study should be interpreted in the context of its strengths and limitations. As a retrospective study, it is not able to assess causality between RT technique and PROs. Furthermore, the use of SABR was rare and therefore our results should be interpreted most broadly as a comparison between simple techniques and more conformal techniques, rather than a comparison in dose difference between SUPR and SABR. In addition, the use of advanced RT techniques was not randomized, and it is highly likely that more fit patients were offered these techniques over simple RT. However, as a large study from a population-based RT program that routinely collects PROs in patients treated for BoM, it is relatively free from selection bias and has broad generalizability.

## 5. Conclusions

In this publicly funded, non-incentivized healthcare system, there were no patient-reported differences in pain or impact of pain on quality of life between simple vs. more conformal advanced RT techniques such as 3DCRT and IMRT. Given that there is increasing utilization of advanced RT techniques in our cohort and other jurisdictions internationally [4,5,18], there is further need for more randomized trials to assess the benefits of these advanced techniques given their increased cost and inconvenience to patients. Patient-reported outcomes should be a primary outcome for future trials looking to palliate patients with BoM.

## Figures and Tables

**Table 1:** Patient characteristics.

Characteristics		Simple RT (*n* = 1623)	Advanced RT (*n* = 89)	*p* Value
Male		56%	64%	0.19
Age	<50	6%	15%	0.02
	50–70	50%	45%	
	>70	44%	40%	
Primary histology	Prostate	27%	28%	0.01
	Breast	20%	16%	
	Lung	20%	11%	
	Gastrointestinal	9%	5%	
	Gynecological	1%	0	
	Head and Neck	3%	6%	
	Lymphoma	11%	14%	
	Non-prostate GU	7%	12%	
	other	3%	9%	
Treatment Region	Spine	55%	51%	<0.001
	Pelvis	21%	27%	
	Upper Extremity	10%	5%	
	Lower Extremity	3%	1%	
	Ribs	8%	6%	
	Skull	1%	5%	
	Other	3%	7%	
Centre	Abbotsford	20%	26%	0.003
	Kelowna	5%	2%	
	Prince George	16%	19%	
	Surrey	17%	2%	
	Vancouver	43%	51%	

**Table 2:** Partial response and complete pain response by patient characteristics.

		Partial Response	*p* Value	Complete Response	*p* Value
Technique binary	Simple RT	50%	0.82	12%	0.99
	Advanced RT	52%		11%	
Technique	Simple RT	50%	0.94	12%	0.97
	3DCRT	57%		13%	
	IMRT	50%		10%	
	SABR	50%		14%	
Gender	Male	49%	0.23	12%	0.65
	Female	52%		13%	
Age	<50	60%	0.08	16%	0.002
	50–70	49%		9%	
	>70	50%		15%	
Primary histology	Prostate	49%	0.05	14%	0.36
	Breast	49%		12%	
	Lung	52%		11%	
	Gastrointestinal	49%		8%	
	Gynecological	59%		23%	
	Head and Neck	28%		9%	
	Lymphoma	57%		15%	
	Non-prostate GU	52%		10%	
	Other	46%		15%	
Treatment region	Spine	50%	0.36	12%	0.47
	Pelvis	50%		12%	
	Upper Extremity	43%		14%	
	Lower Extremity	57%		8%	
	Ribs	51%		14%	
	Skull	39%		8%	
	Other	59%		21%	
Centre	Abbotsford	52%	0.28	13%	0.59
	Kelowna	49%		14%	
	Prince George	48%		9%	
	Surrey	56%		13%	
	Vancouver	48%		12%	

**Table 3:** Multivariable analysis of partial pain response.

Variable		Odds Ratio	95% CI	*p* Value
Technique	Advanced RT (vs. simple RT)	1.001	0.58–1.72	0.99
Gender	Female (vs. male)	1.21	0.88–1.66	0.25
Age	Continuous yearly increments	0.99	0.98–1.00	0.02
Primary histology	Prostate	reference	−	−
	Breast	0.70	0.44–1.13	0.15
	Lung	1.001	0.68–1.47	0.99
	Gastrointestinal	0.79	0.50–1.25	0.31
	Gynecological	1.07	0.38–3.02	0.89
	Head and Neck	0.32	0.14–0.71	0.005
	Lymphoma	1.28	0.84–1.97	0.26
	Non-prostate GU	0.95	0.59–1.53	0.83
	Other	0.63	0.43–1.67	0.63
Treatment region	Spine	reference	−	−
	Pelvis	1.02	0.76–1.37	0.88
	Upper Extremity	0.90	0.60–1.35	0.61
	Lower Extremity	2.78	0.88–8.78	0.08
	Ribs	1.39	0.92–2.11	0.12
	Skull	0.34	0.06–1.80	0.20
	Other	1.85	0.58–1.72	0.09

**Table 4:** Multivariable analysis of complete pain response.

Variable		Odds Ratio	95% CI	*p* Value
Technique	Advanced RT (vs. simple RT)	1.05	0.51–2.14	0.89
Gender	Female (vs. male)	1.16	0.74–1.81	0.52
Age	Continuous yearly increments	1.02	1.00–1.03	0.04
Primary histology	Prostate	reference	−	−
	Breast	0.83	0.44–1.58	0.58
	Lung	0.71	0.42–1.21	0.21
	Gastrointestinal	0.47	0.23–0.99	0.05
	Gynecological	1.42	0.41–4.95	0.58
	Head and Neck	0.66	0.22–2.00	0.47
	Lymphoma	0.99	0.57–1.73	0.97
	Non-prostate GU	0.67	0.34–1.32	0.25
	Other	1.21	0.51–2.86	0.67
Treatment region	Spine	reference	−	−
	Pelvis	1.28	0.86–1.91	0.12
	Upper Extremity	1.53	0.92–2.56	0.11
	Lower Extremity	0.89	0.19–4.06	0.88
	Ribs	1.38	0.80–2.27	0.24
	Skull	0.99	0.12–4.27	0.99
	Other	1.94	0.51–2.14	0.10

**Table 5:** Univariable and multivariable comparison of pain interference on QoL by simple vs. advanced RT technique for BoM.

Interference of Pain On	Univariable Analysis: Mean (SD) Difference Pre vs. Post				
	Simple RT	Advanced RT	3DCRT	IMRT	SABR
General activity	−2.1 (3.7)	−2.2 (3.5)	−2.6 (3.4)	−2.1 (3.7)	−1.6 (2.2)
Mood	−1.8 (3.4)	−2.1 (2.5)	−3.8 (2.7)	−1.2 (2.1)	−1.0 (1.4)
Walking ability	−2.1 (3.7)	−2.0 (3.9)	−2.1 (3.7)	−1.5 (4.8)	−1.5 (2.1)
Normal work	−2.1 (3.8)	−2.3 (4.4)	−3.9 (3.3)	−1.1 (5.2)	−2.5 (3.5)
Relationships	−1.4 (3.5)	−0.9 (3.2)	−1.6 (3.5)	−0.2 (3.4)	−1.0 (1.4)
Sleep	−2.2 (3.5)	−2.3 (3.7)	−4.3 (2.6)	−0.9 (4.1)	−2.5 (2.1)
Enjoyment of life	−2.2 (3.7)	−2.6 (4.3)	−3.8 (3.0)	−2.0 (5.3)	−1.0 (1.4)

## Data Availability

The data presented in this study are available on request from the corresponding author.
